# Detection of protein complexes from affinity purification/mass spectrometry data

**DOI:** 10.1186/1752-0509-6-S3-S4

**Published:** 2012-12-17

**Authors:** Bingjing Cai, Haiying Wang, Huiru Zheng, Hui Wang

**Affiliations:** 1 School of Computing and Mathematics, Computer Sciences Research Institute, University of Ulster, N. Ireland, BT37 0QB, UK

## Abstract

**Background:**

Recent advances in molecular biology have led to the accumulation of large amounts of data on protein-protein interaction networks in different species. An important challenge for the analysis of these data is to extract functional modules such as protein complexes and biological processes from networks which are characterised by the present of a significant number of false positives. Various computational techniques have been applied in recent years. However, most of them treat protein interaction as binary. Co-complex relations derived from affinity purification/mass spectrometry (AP-MS) experiments have been largely ignored.

**Methods:**

This paper presents a new algorithm for detecting protein complexes from AP-MS data. The algorithm intends to detect groups of prey proteins that are significantly co-associated with the same set of bait proteins. We first construct AP-MS data as a bipartite network, where one set of nodes consists of bait proteins and the other set is composed of prey proteins. We then calculate pair-wise similarities of bait proteins based on the number of their commonly shared neighbours. A hierarchical clustering algorithm is employed to cluster bait proteins based on the similarities and thus a set of 'seed' clusters is obtained. Starting from these 'seed' clusters, an expansion process is developed to identify prey proteins which are significantly associated with the same set of bait proteins. Then, a set of complete protein complexes is derived. In application to two real AP-MS datasets, we validate biological significance of predicted protein complexes by using curated protein complexes and well-characterized cellular component annotation from Gene Ontology (GO). Several statistical metrics have been applied for evaluation.

**Results:**

Experimental results show that, the proposed algorithm achieves significant improvement in detecting protein complexes from AP-MS data. In comparison to the well-known MCL algorithm, our algorithm improves the accuracy rate by about 20% in detecting protein complexes in both networks and increases the F-Measure value by about 50% in Krogan_2006 network. Greater precision and better accuracy have been achieved and the identified complexes are demonstrated to match well with existing curated protein complexes.

**Conclusions:**

Our study highlights the significance of taking co-complex relations into account when extracting protein complexes from AP-MS data. The algorithm proposed in this paper can be easily extended to the analysis of other biological networks which can be conveniently represented by bipartite graphs such as drug-target networks.

## Background

Protein-protein interactions (PPIs) are believed to be fundamental to the biological process and metabolic functions in the cell [1]. As advance in high throughput experimental methods and computational approaches, such as Yeast two-hybrid (Y2H) screening [2,3] and Affinity purification/mass spectrometry (AP-MS) [4-6], large genome-scale protein interactions have been detected, resulting in increasing size of PPI networks. Research on PPIs in biology and medicine has shown that a protein complex is a typical pattern existing in PPI networks in which a group of proteins interact with each other to play a biological function in a cell, such as anaphase-promoting complex and protein export and transport complexes [7], or bind each other in a series of time in a biological process such as the yeast pheromone response pathway and Mitogen-activated protein (MAP) signalling cascades [7]. Hence, to identify the group of functionally interacted proteins could help to reveal and understand the relationship between the organization of a network and its function.

Over the past decade or so, various clustering algorithms [7-16] have been proposed for identifying protein complexes in PPI networks. Markov Cluster Algorithm (MCL) [12,13] has been one of the most successful clustering methods in identifying complexes from protein interaction networks. It simulates a flow on the graph by calculating successive power of the associated adjacency matrix. A coefficient called inflation is applied to enhance the contrast between regions of strong and weak flows in the graph. The process converges towards a partition of the graph, with a set of high-flow regions (the clusters) separated by boundaries with no flow. In 2006, Brohée and Helden [17] evaluated four clustering algorithms for their ability to detect protein complexes, and results highlighted that MCL was remarkably robust to graph alterations. Another well-known clustering algorithm is CFinder [11]. It was developed in 2006 based on the idea that a cluster consists of a number of k-cliques where two adjacent *k*-cliques share *k*-1 nodes. It exploits the topological feature of the network by using the direct link between a pair of nodes.

Most of these algorithms have been developed by modelling protein interactions as binary, i.e., interactions only exist between pairs of proteins. Results from the Y2H approach are inherently modelled as binary since the Y2H approach detects physical pair-wise protein-protein interactions. Although AP-MS data contains non-binary information, as it directly identifies co-membership of complexes by purifying proteins (called prey) that are associated with tagged proteins which were used as bait [4-6], it also has been modelled as binary networks where purification is seen as direct pair-wise interactions from bait to its associated prey proteins.

Two well-known binary models for AP-MS data are 'Spoke' and 'Matrix' models which have been proposed in 2003 by Bader and Hogue [8]. The 'Spoke' model is similar to a 'Star' topology where bait proteins are the "hub" nodes and purified prey proteins are connected with baits. 'Matrix' model is in the other extreme, that is, besides associate interactions between prey proteins and bait proteins, all these prey proteins are all connected as well. 'Matrix' model for a complex is actually a 'clique' structure. The real topology of the set of proteins lies between these two models [8]. The Molecular Complex Detection (MCODE) algorithm has been developed for identifying densely connected sections of a PPI networks. It weighs proteins by the density of their neighbourhood and takes proteins with highest weight as seeds of clusters. Starting from these seeds, MCODE expands clusters in the network in a greedy fashion. It has been evaluated using Gavin data set [4] by treating it as 'Spoke' model.

In 2006, Gavin et al., [5] devised a 'socio-affinity' scoring system to weigh logical interactions between pairs of proteins in AP-MS data. In this study, several clustering methods have been employed to cluster on the scored PPI networks. In 2007, Collins et al. [18] developed another scoring system and applied hierarchical clustering methods to weighted networks to derive complexes. Afterwards, Pu et al., [19] applied MCL on the scoring system of Collins et al. [18] to detect protein complexes.

The study of Gavin et al. [5] highlighted that a protein complex generally contains a core in which proteins are highly co-expressed and share high functional similarity. The COACH approach was proposed in 2009 [20], aiming at detecting protein complexes with highly-dense structure as well as exploring "core-attachment" organization inside protein complexes. The process of extracting protein complexes by COACH [20] consists of two stags. Firstly COACH [20] generates neighbourhood graphs of every node from the original network and then extracts preliminary set of core complexes which are of high density from each neighbourhood graphs. After a redundancy-filtering procedure, a set of final core complexes is obtained. In the second stage, an expansion process is conducted by exploring periphery information of cores to find attachments which consist of complete protein complexes.

The first study of modelling AP-MS data as non-binary was conducted by Scholtens et al., [21]. They built the spoke model of AP-MS data as a directed network where edges link from bait proteins to prey proteins, and then the Local Modelling algorithm [21] was applied to this directed network. Results showed that predicted clusters from the Local Modelling algorithm well mapped curated protein complexes.

Most recently, in 2011, a novel algorithm called CODEC [22] has been proposed to cluster AP-MS data. CODEC translated AP-MS data to a bipartite graph, where all proteins in the network are classified into two sets, 'Baits' and 'Preys', and interactions only exist between these two sides. CODEC method aims to detect complexes as dense bipartite sub-graphs. It has been applied to three PPI networks of Yeast [4,5,23]. Results showed the CODEC method outperformed other algorithms with higher precision.

As pointed out by Geva and Sharan [22], AP-MS data could be directly applied for identifying complexes since AP-MS experiments detect complex co-membership. Modelling it as a bipartite graph could be more fitted to the non-binary nature of AP-MS data. Preserving information of bait protein when AP-MS data is modelled may help to improve the accuracy of identifying and predicting protein complexes and functional modules.

This paper presents a novel algorithm for detecting protein complexes from AP-MS data. The algorithm intends to detect groups of prey proteins that are significantly co-associated with the same set of bait proteins. We first construct AP-MS data as bipartite network, where one set of nodes consists of bait proteins and the other set is composed of prey proteins. We then calculate pair-wise similarities of bait proteins based on the number of their commonly shared neighbours. A hierarchical clustering algorithm is employed to cluster bait proteins based on the similarities and thus a set of 'seed' clusters is obtained. Starting from these 'seed' clusters, an expansion process is developed to identify prey proteins which are significantly associated with the same set of bait proteins. Then, a set of completely formed protein complexes are derived.

The organization of the paper is shown below. In Section 2, we first introduce the methodology of our proposed algorithm. In Section 3, we will present and discuss experimental results. We validate biological significance of predicted protein complexes by using curated complexes and well-characterized cellular component from GO [24]. Several statistical metrics have been applied for evaluation. The paper is concluded with conclusion and the discussion of the limitation and future work.

## Methods

The AP-MS experiment directly detects complex membership by purifying prey proteins which are co-associated with tagged bait proteins [4,5]. Thus, an assumption of protein complexes can be derived, that, in AP-MS data, a complex is composed of a set of bait proteins along with a set of prey proteins that are significantly associated with the same set of bait proteins. Our proposed method is developed based on this assumption.

**Figure 1:**
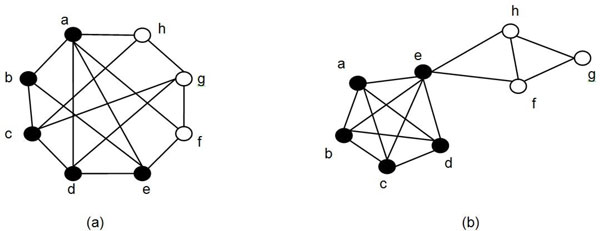
**Two typical graphs of the same size and density, but different topological structure**. Figure 1 shows two graphs which contain the same number of nodes and edges and has the same density, but they have different topological structure.

Though protein complexes are considered to contain proteins having most similarities, there is no one standard definition of protein complexes from the perspective of topological structure. A set of proteins which are highly connected is intuitively considered as a complex, e.g., a clique is an ideal structure for a complex. However, ensuring density of internal interactions only is not enough to identify a complex, as discussed in [25]. Figure 1[25] shows the two typical graphs of the same size and density, which have the same density but different topological structure. Different topological structures that may represent a complex exist in real PPI networks. A 'Star' shape where all nodes connected to a 'hub' protein is an example. Mixture of a clique and a 'Star' shape complex is also possible, leading to more complex topologies. Thus, by considering the basic heuristic that interactions between nodes inside the same cluster are more than those that link to outside, we employ definitions of 'strong module' and 'weak' module proposed by Radicchi et al., [26], to define protein complexes.

Here, a module is referred to as a term of a complex structure. In a strong module each vertex has more connections with the cluster than with the rest of the graph. In a weak module, the sum of all links connecting from each node within the cluster is larger than the sum of all links connecting from each node inside the cluster toward the rest of the network. In PPI networks, there exist complexes which have structure of a strong module, or of a weak module, or a combination of the two. However, all complexes should meet requirements in the definition of weak modules.

We represent AP-MS data as a bipartite graph. The graph is denoted as G=(B,V,E), where *B *represents the set of purification with bait nodes which is on the one side, *V *represents the set of prey nodes on the other side that have been detected by purifying via the bait nodes. If let P  be the original set of preys that is obtained directly from the dataset, V=B∪P. Thus, *V *is the union set of bait nodes and prey nodes, which, in other words, *V *is the set of nodes in the network. Note that, there exist nodes that are preys of some baits but also baits to other preys, thus we assign them a bait instance and prey instance respectively. *E *represents pair-wise interactions between baits and preys. A potential protein complex or functional module corresponds to a sub-graph $G^{\prime }=\left(B^{\prime },V^{\prime },E^{\prime }\right)$ of the graph, where $V^{\prime }\subseteq V$ is the set of nodes in the cluster, and $B^{\prime }\subseteq B$ is the set of corresponding baits.

**Figure 2:**
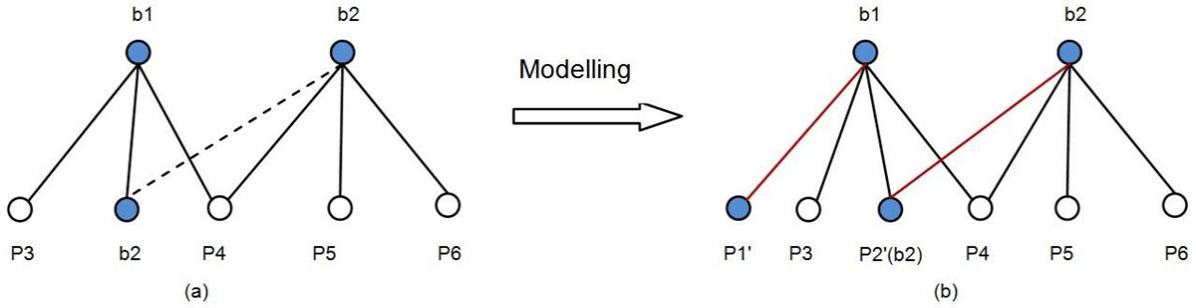
**Model AP-MS data as bipartite graph**. Figure 2 demonstrate the process of modelling AP-MS data as bipartite graph.

Figure 2 shows the modelling process. Figure 2-(a) represents the original graph, where $b_{1}$and $b_{2}$are bait nodes and $p_{3}$, $p_{4}$, $p_{5}$, $p_{6}$are prey nodes. Note that, $b_{2}$ is also a prey of $b_{1}$. Figure 2-(b) is the bipartite graph model we built from the original graph. As described above, we add a prey instance for each bait protein.

The process of detecting complexes in our proposed algorithm consists of the following steps:

1. Calculating pair-wise similarities between bait proteins;

2. Clustering bait proteins to obtain preliminary seed clusters;

3. Expanding process to form complete clusters;

4. Filtering clusters and outputting final set of clusters.

### Calculating pair-wise similarities between bait proteins

We estimated pair-wise similarity between two baits based on the number of their common neighbours using *Jaccard Similarity Coefficient *[27]. Let $b_{1}$ and $b_{2}$ be the two baits, $N\left(b_{1}\right)$ and $N\left(b_{2}\right)$ denote the set of neighbours of $b_{1}$ and $b_{2}$, respectively. Then $sim\left(b_{1},b_{2}\right)$ is:

(1) $$sim\left(b_{1},b_{2}\right)=\frac{\left|N\left(b_{1}\right)\cap N\left(b_{2}\right)\right|}{\left|N\left(b_{1}\right)\cup N\left(b_{2}\right)\right|}$$

We calculate the similarity between every pair of bait proteins in the graph. Thus, let E(sim(B)) be the set of values of similarity between pairs of baits, and then form a network based on these similarities, that is, $G\left(B\right)=\left(B,E\left(sim\left(B\right)\right)\right)$, where *B *represents the set of bait proteins.

### Clustering bait proteins to obtain preliminary seed clusters

In order to identify the set of prey proteins that are significantly associated with the same set of bait proteins, we first need to obtain sets of bait proteins as 'seed clusters'. Using the similarities calculated above as metric, we apply Agglomerative Hierarchical Cluster algorithm to cluster the bait proteins. We employ an open source tool called MultiDendrograms [28] to clusterbait proteins. MultiDendrograms [28] incorporates most common Agglomerative Hierarchical Clustering algorithms, e.g. Single Linkage, Complete Linkage and Unweighted Average. Selection of parameters in experiments will be introduced in the following result section.

### Expanding process to form complete clusters

As shown in the work published by Gavin et al., [4], a protein complex generally contains a core in which proteins are highly co-expressed and share high functional similarity. Some protein cores are surrounded by attachments which help supporting subordinate functions. Inspired by the finding, we consider that the cores correspond to the structure of a strong module [26] we introduced above and the attachment corresponds to the structure of a weak module [26]. A complete cluster should meet the requirements of a weak module. Thus, the expansion process is composed of two stages: firstly, detecting strong modules from seed clusters which are composed of bait proteins only; secondly, expanding to form final clusters from the strong modules of clusters in a greedy fashion.

#### 1. Detect strong modules from seed clusters

Let *Sc *be a seed cluster, and let *u *be a prey protein connecting with proteins in the seed cluster. Let $d_{in}\left(u,Sc\right)$ be the number of connections from *u *to *Sc*; $d_{out}\left(u,Sc\right)$ be the number of connections of *u *to proteins that are not in *Sc*; let $C_{in}\left(Sc+u\right)$ be the number of internal connections inside the cluster in which *u *is included in *Sc*; $C_{out}\left(Sc+u\right)$ be the number of external edges from the cluster in which u is included in *Sc*.

We start from the neighbourhood of bait proteins in the seed cluster. If a prey protein is able to be included into the strong module, it should satisfy:

1) *u *should connect to at least half proteins inside the seed cluster, that is,

(2) $$d_{in}\left(u,Sc\right)\geq 0.5*|Sc|$$

where |Sc| is the size of the seed cluster *Sc*.

2) The connections from *u *to proteins inside the seed cluster should be more than connections linking to other proteins which are not in the seed cluster, that is,

(3) $$d_{in}\left(u,Sc\right)>d_{out}\left(u,Sc\right)$$

3) The out-links of seed cluster which includes *u *should be less than the internal links, that is,

(4) $$C_{out}\left(Sc+u\right)<C_{in}\left(Sc+u\right)$$

The process ceases when there is no matched protein.

#### 2. Form final clusters

After the expansion process of finding strong modules, the process of forming final clusters starts. It will iteratively explore matched proteins in the neighbourhood of the proteins in strong modules.

Suppose *v *is a candidate protein, *Mc *is the strong module cluster after expansion from *Sc*, *v *should meet following criteria:

1) The connections from *v *to proteins in *Mc *should be no less than those from *v *to other proteins, that is,

(5) $$d_{in}\left(v,Mc\right)\geq d_{out}\left(v,Mc\right)$$

2) After included *v*, the internal links inside the new cluster should be more than the external links, that is,

(6) $$C_{in}\left(Mc+v\right)>C_{out}\left(Mc+v\right)$$

Actually, there exist "seed clusters" only consist of one bait protein; we just add its neighbours if the neighbour protein meets the two conditions above.

### Filtering clusters and outputting final set of clusters

In the set of clusters obtained from expansion process, there exist overlapping clusters. We calculate the overlap rate between two clusters, that is, $\left(\left|C_{1}\cap C_{2}\right|\right)/\left(\left|C_{1}\cup C_{2}\right|\right)$, where *|C| *is the size of the cluster. If the overlap rate is above a given threshold, we merge the two clusters. In our algorithm, we use 0.2 as the threshold value.

### Time complexity

The general time complexity of the entire algorithm is $O\left(m^{2}+m^{3}+kmn+h^{2}\right)$, where m  represents the number of bait instances in the network and n  is the number of prey instances which is also the size of the network (when modelling the network we add instances of bait proteins to prey instances side.), m<n. h  represents the number of predicted clusters obtained. The first step of our algorithm is to calculate pair-wise similarities between bait nodes, thus the time complexity is $O\left(m^{2}\right)$. In the second step, the time complexity for agglomerative hierarchical algorithm is $O\left(m^{3}\right)$. As for expansion process, the time complexity of one expansion process is O(mn). Since we adopt greedy fashion in expansion, there may by *k *times of expansion, thus the time complexity for the whole expansion process is O(kmn). The post-process stage could be up to $O\left(h^{2}\right)$. Normally, since $n<m^{2}$ and h<m, thus, the asymptotic time complexity of our algorithm is $O\left(m^{3}\right)$.

### Implementation and running time

We implemented the proposed method using Java programming language with JDK 1.6. The proposed method is applied on a desktop computer with Inter(R) Core(TM)2 Duo CPU E8500 @3.16GHz 3.17 GHz processor and 8 GB memory. The amount of running time depends on the size of dataset. The running time of the proposed method on Gavin_2006 dataset was 34653 milliseconds, and the application to Krogan_2006 dataset was 61336 milliseconds. This running time only contained the time of process of calculation of pair-wise similarity for bait proteins, expansion process and post-process, excluding the time of application of hierarchical clustering method to generate seed clusters since we utilized the software toolkit, MultiDendrograms [28], for this purpose.

## Results

### Preparation of data

We applied our method on two recently published datasets in bait-prey relationships in Yeast. One is the dataset obtained by Gavin et al [5] with 1993 bait proteins, 2671 prey proteins and 19157 bait-prey relationships; the other is the dataset published by Krogan et al. [23], which contains 2233 bait proteins, 5219 prey proteins and 40623 bait-prey relationships. 94 prey proteins were suspected as non-specific contaminants [23] so that they were excluded from the raw data of Krogan et al's dataset. For convenience, we name these two datasets as Gavin_2006 and Krogan_2006 for short.

We built the benchmark set of complexes from hand-curated complexes derived from the Wodak lab CYC2008 catalogue [29] which contains 408 complexes. In order to evaluate the biological coherence of our predicted complexes, we also download the list of cellular localizations (GO terms under "Cellular Component") of proteins from GO [24]. Specifically, we derived complexes with Cellular Component annotations below the "protein complex" GO term. We remove GO annotations with *IEA *evidence codes due to their lack of reliability. Thus, a total of 319 protein complexes were derived accordingly. To fairly evaluate performance of different methods, we only considered the set of benchmark complexes that contain at least 2 proteins which are in PPI networks. We employ similar method of pre-processing benchmark complexes in literatures [22,30], that is, we exclude complexes which have number of overlapped proteins with those in networks less than 2. Thus, a final set of benchmark complexes is generated for each PPI network. Table 1 shows figures of number and average size of known complexes derived from the two testing PPI networks. For convenience, we call these two sets of benchmark complexes CYC-2008 and GO-CC for short.

**Table 1:** The number and average size of known complexes derived from two PPI networks

PPI networks	Gavin_2006		Krogan_2006	
	CYC-2008	GO-CC	CYC-2008	GO-CC
No. of complexes	360	283	406	311
Ave. size	5.00	5.83	4.72	5.55

### Quality assessment

We utilize evaluation metrics, i.e., accuracy and homogeneity suggested by Broheé and Helden [17]. These two metrics measures the overlap degree between predicted clusters and benchmark complexes.

Let C  be the set of predicted clusters generated by the clustering algorithm, and let $C^{*}$ be the subset of C , $C^{*}\subseteq C$, containing clusters that have at least two nodes annotated in any of benchmark complexes. Let M  be the set of benchmark complexes and let $M^{*}$, $M^{*}\subseteq M$, be the set of benchmark complexes excluding those which contain proteins that are not found in the network. Let n  be the number of clusters in C*, and m  be the number of complexes in $M^{*}$, then a n×m confusion matrix Z  is constructed for comparison between predicted clusters and benchmark complexes. The *i^th ^*row stands for predicted cluster i  while the $j^{th}$ column corresponds to benchmark complex j . The entry $z_{ij}$ represents the number of proteins found in $i^{th}$ cluster that are annotated in $j^{th}$ benchmark complex. $z_{i}$ is the size of $i^{th}$ predicted cluster while $z_{j}$ represents size of $j^{th}$ benchmark complex.

#### • Accuracy

Accuracy measures the general correspondence between predicted clusters and benchmark complexes, which contains two components, sensitivity (Se) and positive predictive value (PPV).

Sensitivity is defined to calculate the proportion of proteins in benchmark complex j  which are covered in predicted cluster i . The maximal sensitivity value of benchmark complex j  is obtained which indicates the coverage of the $j^{th}$ by its best-matching predicted cluster. The general sensitivity is the weighted average of each benchmark complex's best sensitivity value over all benchmark complexes,

(7) $$Se=\frac{\sum_{j=1}^{m}\left(z_{j}\cdot max_{j=1}^{m}\frac{z_{ij}}{z_{j}}\right)}{\sum_{j=1}^{m}z_{j}}$$

PPV reveals the fraction of proteins annotated in benchmark complex j  in a predicted cluster i . Again, the maximal PPV of each predicted cluster is calculated. The general positive predictive value can be computed over all the clusters as the weighted average of each predicted cluster's best PPV,

(8) $$PPV=\frac{\sum_{i=1}^{n}z_{i}\cdot max_{i=1}^{n}\frac{z_{ij}}{\sum_{j=1}^{m}z_{ij}}}{\sum_{i=1}^{n}z_{i}}$$

Accuracy is defined as the geometric mean of the product of general sensitivity and positive predictive value,

(9) $$Accuracy=\sqrt{Se\times PPV}$$

Thus, high precision value requires a high performance for both measures. The higher precision values the better quality of a clustering result.

#### • Homogeneity

Complex-wise homogeneity $hM_{j}$ shows the fraction of proteins in a same benchmark complex *j *over all the generated clusters. Meanwhile, cluster-wise homogeneity $hC_{i}$ is defined to represent the distribution of proteins detected as members in the same cluster *i *over annotated complexes. These two measures can be represented as:

(10) $$hM_{j}=\overset{n}{\underset{i}{\sum }}\left(\frac{z_{ij}}{\sum_{i=1}^{n}z_{ij}}\times \frac{z_{ij}}{\sum_{j=1}^{m}z_{ij}}\right)$$

(11) $$hC_{i}=\overset{m}{\underset{i}{\sum }}\left(\frac{z_{ij}}{\sum_{j=1}^{m}z_{ij}}\times \frac{z_{ij}}{\sum_{i=1}^{n}z_{ij}}\right)$$

Similarly, in order to reflect a clustering result as a whole, the average values of $hM_{j}$ and $hC_{i}$ is calculated respectively and shown as follows:

(12) $$meanhM=\frac{1}{m}\times \overset{m}{\underset{j=1}{\sum }}hM_{j}$$

(13) $$meanhC=\frac{1}{n}\times \overset{n}{\underset{i=1}{\sum }}hC_{i}$$

Thus clustering-wise homogeneity Ho is defined as the geometric mean of the product of general meanhM and meanhC,

(14) $$Ho=\sqrt{meanhM\times meanhC}$$

Homogeneity reflects relative ratio of distribution of overlapping intersections between annotated complexes and generated clusters. When proteins are allowed to be assigned to multiple clusters, the value $hC_{i}$ will be lower and thus the homogeneity value will be lower.

#### • Sensitivity and specificity

To further assess the quality of predicted clusters, we also measure the specificity and sensitivity of predicted complexes with respect to the set of benchmark complexes. We utilize the overlap score introduced by Bader and Hogue [8] to measure the level of significant match of a predicted cluster $C_{i}$, with regard to a known complex $M_{j}$, that is, $\rho =\left(\left|C_{i}\cap M_{j}\right|\right)/\left(\left|C_{i}|*|M_{j}\right|\right)$, where $\left|C_{i}\cap M_{j}\right|$ is the number of overlapped proteins between the predicted cluster $C_{i}$ and the known complex $M_{j}$, $\left|C_{i}|*|M_{j}\right|$ is the product of the size of the predicted cluster and the known complex. For each predicted cluster, we identify a known complex with which the intersection is the most significant according to a threshold value of ρ . Thus, according to the analysis of a specificity and sensitivity in [8], the number of true positives (TP) is defined as the number of predicted clusters with the value of ρ  over a given threshold and the number of false positives (FP) is defined as the total number of predicted clusters minus TP. The number of false negatives (FN) is the number of benchmark complexes which are not matched by any predicted complexes. Thus,

(15) Sensitivity=TP/(TP+FN)

and

(16) specificity=TP/(TP+FP)

F-Measure is the harmonic average of sensitivity and specificity. In our experiments, based on the study of Bader and Hogue [8], we consider that a predicted cluster significantly matches a benchmark complex if the corresponding ρ≥0.2.

### Selection of parameters

We selected the parameters following a trial-and-error procedure. Unless indicated otherwise, the results reported in this paper were derived based on the following parameter settings: the hierarchical clustering was implemented with un-weighted average linkage and the cut-off values set to 0.3 and 0.25 for Gavin_2006 and Krogan_2006 networks, respectively.

We choose the set of parameters of MCL and MCODE recommended by Broheé and Helden [17]. Specifically, we use inflation rate 1.8 for MCL. For MCODE, we set the parameters depth equal to 100, node score percentage as 0, Haircut is TURE, Fluff is FALSE and the percentage for complex fluffing as 0.2. The k  value required for CFinder was set to 5. As for the CODEC algorithm, there are two schemes, CODEC-w0 and CODEC-w1. We compare our method to both schemes of CODEC. We only use final predicted clusters from COACH, without considering its predicted core clusters.

### Experimental results and discussion

We compare performance of our method with that of several state-of-art clustering methods, which are categorized into two groups. One includes MCL [12,13], MCODE [8], CFinder [11], and COACH [20], each treating AP-MS data as non-bipartite graph; and the other is CODEC [22] the algorithm that treated AP-MS data also as a bipartite graph. The input for algorithms in the first category is the set of interactions from a bait protein to its preys represented as the Spoke model [8].

#### • Accuracy and homogeneity

Evaluation results of accuracy and homogeneity, using benchmark complexes from CYC-2008 catalogue and GO-CC, of different clustering methods in Gavin network are listed in Tables 2 and 3.

**Table 2:** Performance comparison on Gavin_2006 with CYC-2008

PPI networks	Gavin_2006						
Evaluation Metric	MCL	MCODE	CFinder	COACH	CODEC-w0	CODEC-w1	Our method
**Sensitivity**	**0.721**	0.338	0.390	0.596	0.584	0.582	0.434
**PPV**	0.296	0.342	0.365	0.120	0.511	0.546	**0.747**
**Accuracy**	0.462	0.340	0.377	0.268	0.546	0.564	**0.570**
**Complex-wise Homogeneity**	0.156	0.123	0.087	0.048	0.234	0.272	**0.318**
**Cluster-wise Homogeneity**	**0.875**	0.748	0.613	0.030	0.086	0.107	0.848
**Homogeneity**	0.369	0.303	0.231	0.038	0.141	0.171	**0.519**

**Table 3:** Performance comparison on Gavin_2006 with GO-CC

PPI networks	Gavin_2006						
Evaluation Metric	MCL	MCODE	CFinder	COACH	CODEC-w0	CODEC-w1	Our method
**Sensitivity**	**0.655**	0.333	0.358	0.549	0.524	0.529	0.420
**PPV**	0.241	0.337	0.335	0.107	0.314	0.405	**0.648**
**Accuracy**	0.397	0.335	0.346	0.242	0.406	0.463	**0.522**
**Complex-wise Homogeneity**	0.164	0.140	0.087	0.050	0.249	0.288	**0.350**
**Cluster-wise Homogeneity**	**0.845**	0.746	0.536	0.027	0.098	0.123	0.800
**Homogeneity**	0.373	0.323	0.216	0.036	0.156	0.188	**0.529**

The results shown in Table 2 highlight that the proposed method achieves the highest PPV and Accuracy value, although it has relatively low sensitivity. Sensitivity measures how well a complex could be found in the cluster. We could see from Tables 2, 3, 4 and 5, MCL has the highest sensitivity value and COACH and CODEC achieve relatively higher sensitivity as well on both Gavin_2006 and Krogan_2006 network. This could be partially attributed to the inclusion of larger modules in their results. For example, as shown in Table 6, COACH tends to yield larger clusters since the average size of generated clusters is about 78 and 181 in Gavin_2006 and Krogan_2006 dataset, respectively. A large cluster may be composed of several smaller benchmark complexes so that the sensitivity value of these small benchmark complexes is very high. As for MCL, although the average size of predicted clusters is not the highest, there exists a very large cluster in the clustering result. For instance, tuning the inflation rate from 1.8 to 2.5, the size of biggest cluster from MCL varies from 1578 to 875, which may not be biologically meaningful.

**Table 4:** PerformancecComparison on Krogan_2006 with CYC-2008

PPI networks	Krogan_2006						
Evaluation Metric	MCL	MCODE	CFinder	COACH	CODEC-w0	CODEC-w1	Our method
**Sensitivity**	0.659	0.275	0.346	**0.660**	0.595	0.562	0.300
**PPV**	0.140	0.135	0.389	0.076	0399	0.422	0.550
**Accuracy**	0.304	0.193	0.366	0.224	**0.487**	**0.487**	0.406
**Complex-wise Homogeneity**	0.052	0.036	0.063	0.015	**0.232**	0.218	0.134
**Cluster-wise Homogeneity**	0.537	0.474	0.566	0.003	0.024	0.048	**0.745**
**Homogeneity**	0.373	0.323	0.216	0.036	0.156	0.188	**0.529**

**Table 5:** Performance comparison on Krogan_2006 with GO-CC

PPI networks	Krogan_2006						
Evaluation Metric	MCL	MCODE	CFinder	COACH	CODEC-w0	CODEC-w1	Our method
**Sensitivity**	0.644	0.274	0.340	**0.638**	0.543	0.517	0.303
**PPV**	0.102	0.126	0.354	0.060	0.350	0.361	**0.443**
**Accuracy**	0.257	0.186	0.347	0.196	0.436	**0.432**	0.366
**Complex-wise Homogeneity**	0.049	0.039	0.076	0.015	0.241	**0.225**	0.138
**Cluster-wise Homogeneity**	0.450	0.469	0.511	0.002	0.026	0.046	**0.729**
**Homogeneity**	0.149	0.136	0.197	0.006	0.079	0.102	**0.317**

**Table 6:** Number and average size of predicted clusters from different methods on the two testing PPI networks (exclude singleton clusters)

	MCL	MCODE	CFinder	COACH	CODEC-w0	CODEC-w1	Our method
**Gavin_2006**							
**No. of clusters**	223	100	65	612	1082	1005	461
**Ave. size**	11.5	12.1	16.4	78.1	17.3	13.8	5.1
**Krogan_2006**							
**No. of clusters**	379	73	73	1927	8348	2973	588
**Ave. size**	14.1	25.2	15.1	181.8	16.1	16.2	5.0

PPV value indicates the fraction of clustering results which have also been identified and annotated in the benchmark complexes so far. It favours smaller clusters. In order to be fair, as stated above, we excluded clusters whose size of overlap with curated complexes is less than two proteins. Results show that our method obtains the highest PPV value in comparison to other algorithms. The better accuracy suggests that the proposed algorithm can achieve a much better performance as the value of the accuracy reflects the general performance of a clustering algorithm based on the estimation of the overall correspondence between the set of predicted clusters and the set of annotated complexes.

Homogeneity is the product of the fraction of members in a cluster found in an annotated complex by the fraction of members in the complex found in a cluster. High homogeneity indicates a bi-directional correspondence between a cluster and a complex [17]. The maximal value of homogeneity is 1 when a cluster matches perfectly with a complex which means that the cluster consists of all its members identified in the complex. As shown in Table 2, the proposed algorithm achieves the best performance in terms of the clustering-wise homogeneity value, which reflects the general agreement between identified clusters and benchmark complexes, as well as the quality of a clustering result as a whole.

Similar observations can be made when analysing the Krogan_2006 data as shown in Tables 4 and 5. Our proposed method outperforms other clustering algorithms except CODEC. While CODEC_w1 yields better accuracy than our proposed method, it yields a very low value of homogeneity. This could partly be due to the high level of overlap between clusters generated. In the clustering results obtained by using CODEC-w1, the average overlap rate between predicted clusters is 52% and 50% for Gavin_2006 and Krogan_2006 datasets, respectively. Though relatively lower accuracy value than CODEC-w1, our proposed method still achieves the best performance in terms of both highest PPV and homogeneity.

#### • Specificity and sensitivity

Table 7 shows the result of specificity and sensitivity for clustering results produced by each clustering algorithm when apply to Gavin_2006 network. As discussed in the previous section, the sensitivity is used to measure the percentage of benchmark complexes recovered by predicted clusters whose overlap score satisfies the given threshold. As shown in Table 7, the sensitivity derived from all the algorithms are very high for Gavin_2006 dataset, suggesting that almost all benchmark complexes in the derived set are recovered by predicted clusters. Nevertheless, the proposed method achieves the highest specificity value which measures fraction of predicted clusters that match benchmark complexes. This suggests our proposed method reaches a much higher level of true positives in the clustering results. Moreover, the proposed algorithm achieves the highest F-Measure further demonstrating its performance.

**Table 7:** Specificity/sensitivity/F-measure results on the two testing PPI networks with CYC-2008 and GO-CC benchmark complexes on Gavin_2006

	MCL	MCODE	CFinder	COACH	CODEC-w0	CODEC-w1	Our method
**CYC_2008**							
**Specificity**	0.859	0.610	0.804	0.252	0.305	0.459	**0.889**
**Sensitivity**	1.000	1.000	1.000	1.000	1.000	1.000	**1.000**
**F-Measure**	0.924	0.758	0.891	0.403	0.468	0.630	**0.941**
**GO_CC**							
**Specificity**	0.836	0.585	0.761	0.197	0.385	0.533	**0.839**
**Sensitivity**	0.939	0.456	0.897	0.963	0.993	0.994	**0.963**
**F-Measure**	0.885	0.512	0.824	0.328	0.555	0.694	**0.897**

In the results obtained from Krogan_2006 network (shown in Table 8), CFinder achieves highest specificity value, but its sensitivity value is lower. MCODE, COACH, as well as MCL, also have lower sensitivity value compared to that obtained in Gavin_2006 network. Both CODEC and our proposed method still recover most fractions of benchmark complexes, highlighting the significance of the incorporation of co-complex relations into the analysis of AP-MS data. However, the proposed method achieves the highest F-measure which again indicates our proposed method outperforms other clustering algorithms in terms of the overall performance measurement.

**Table 8:** specificity/sensitivity/F-measure results on the two testing PPI networks with CYC-2008 and GO-CC benchmark complexes on Krogan_2006

	MCL	MCODE	CFinder	COACH	CODEC-w0	CODEC-w1	Our method
**CYC_2008**							
**Specificity**	0.333	0.226	**0.800**	0.047	0.324	0.502	0.740
**Sensitivity**	0.520	0.048	0.600	0.822	1.000	1.000	**1.000**
**F-Measure**	0.406	0.080	0.686	0.088	0.489	0.668	**0.850**
**GO_CC**							
**Specificity**	0.265	0.231	**0.739**	0.026	0.298	0.443	0.729
**Sensitivity**	0.900	0.105	0.723	0.685	0.999	0.999	**0.977**
**F-Measure**	0.409	0.145	0.731	0.051	0.458	0.614	**0.835**

#### • Analysis of biological significance of clustering

To further validate biological significance of the results obtained by the proposed method, we next discuss several predicted complexes that are found by our method but not detected by other methods, which are also biological relevant. Here, we present examples of clusters obtained from Krogan_2006 network.

One example of fully-matched clusters identified by the proposed algorithm but not found in results produced by other algorithms, includes four proteins, that is YJR112W, YPL233W, YAL034W-A, and YIR010W. This protein complex has been defined as a kinetochore complex that binds to centromeric chromatin and forms part of the inner kinetochore of a chromosome in the nucleus [31,32]. Another cluster found by our method and not identified by other algorithms, is composed of five proteins, YIL097W, YMR135C, YIL017C, YGL227W and YDR255C, which are all annotated by GO term: 0034657 (GID complex) [33,34]. Although not including all proteins, the predicted complex matches five out of seven proteins in the complex with ubiquitin ligase activity that is involved in proteasomal degradation of fructose-1,6-bisphosphatase (FBPase) and phosphoenolpyruvate carboxykinase during the transition from gluconeogenic to glycolytic growth conditions [33,34]. Another example is the cluster consisting of six proteins, i.e., YJR082C, YEL018W, YNL136W, YFL024C, YOR244W and YHR090. Among these six proteins, three belong to the subunits of NuA4 in baker's yeast, within an essential histone H4/H2A acetyltransferase complex annotated by GO cellular component term GO:0032777 [35,36]. Although not all listed in the protein complex, the other 3 proteins found in the cluster have been identified as subunit of the NuA4 histone acetyltransferase complex in the yeast [35,36].

These cases exemplify that, by the incorporation of information of bait proteins in the clustering analysis of AP-MS data, the propose method has the advantage to discover significant functional modules from the networks.

## Conclusions

In this paper, we propose a new algorithm for discovering functional modules and complexes in AP-MS PPI networks. It has been tested on two real AP-MS PPI networks, i.e., Gavin_2006 [5] network and Krogan_2006 network [23]. Comparing to well-known MCL algorithm, our algorithm improves the accuracy rate by about 20% in extracting protein complexes from both AP-MS networks and increases the F-measure value by about 50% on Krogan_2006 network. Greater accuracy, better homogeneity and higher specificity and sensitivity were achieved in comparison with the results produced by several state-of-art clustering algorithms. The main feature of our method is that it detects protein complexes by taking co-complex relations into account from AP-MS data. Furthermore, the proposed method is able to detect overlapping modules encoding in PPI networks. In addition, the framework proposed in this paper can be easily extended to the analysis of other biological networks which can be conveniently represented by bipartite graphs such as drug-targets networks.

Currently, our proposed algorithm only considers the topological features of PPI networks. Incorporation of other biological information such as semantic similarity derived from GO into the clustering process would be an important part of our future work.

In this study, the determination of the parameters was based on trial and error. Integration with other techniques such as Genetic Algorithm for the dynamic determination of learning parameters provides another direction of our research.

## Competing interests

The authors declare that they have no competing interests.

## Authors' contributions

BC conceived of the study and carried out all programming and analyses as a Ph.D student in the University of Ulster. HYW supervised the study and provided valuable input for experiments. HZ and HW supervised the study and helped to draft the manuscript. All authors read and approved the final manuscript.
